# PhyloMatcher: a tool for resolving conflicts in taxonomic nomenclature

**DOI:** 10.1093/bioadv/vbad144

**Published:** 2023-10-05

**Authors:** Jonathan A Rader, Madelyn A Pivovarnik, Matias E Vantilburg, Logan S Whitehouse

**Affiliations:** Department of Biology, University of North Carolina at Chapel Hill, Chapel Hill, NC, 27599-3280, United States; Department of Biology, University of North Carolina at Chapel Hill, Chapel Hill, NC, 27599-3280, United States; Department of Biology, University of North Carolina at Chapel Hill, Chapel Hill, NC, 27599-3280, United States; Department of Genetics, University of North Carolina at Chapel Hill, Chapel Hill, NC, 27599-7264, United States

## Abstract

**Summary:**

Large-scale comparative studies rely on the application of both phylogenetic trees and phenotypic data, both of which come from a variety of sources, but due to the changing nature of phylogenetic classification over time, many taxon names in comparative datasets do not match the nomenclature in phylogenetic trees. Manual curation of taxonomic synonyms in large comparative datasets can be daunting. To address this issue, we introduce PhyloMatcher, a tool which allows for programmatic querying of the National Center for Biotechnology Information Taxonomy and Global Biodiversity Information Facility databases to find associated synonyms with given target species names.

**Availability and implementation:**

PhyloMatcher is easily installed as a Python package with pip, or as a standalone GUI application. PhyloMatcher source code and documentation are freely available at https://github.com/Lswhiteh/PhyloMatcher, the GUI application can be downloaded from the Releases page.

## 1 Introduction

Large datasets and broad comparative studies have become the research paradigm across multiple disciplines of biology (Stephens *et al.* 2015, da Silva *et al.* 2019, Muñoz and Price 2019, Yu and Nielsen 2019, Pal *et al.* 2020, Wüest *et al.* 2020, Xia *et al.* 2020, Tolani *et al.* 2021). The rise of -omics technologies and high-throughput data collection permit rapid collection of data reflecting a wide variety of physiological, morphological, behavioral, and ecological traits (e.g. Nilsson *et al.* 2019, Li *et al.* 2020, Modahl *et al.* 2020, Chen *et al.* 2021). Genomic methods and the decreasing cost of sequencing have also made it possible to assemble large-scale phylogenies that will increasingly facilitate comparative exploration of trait data to illuminate macroevolutionary and macroecological patterns (e.g. Buckley *et al.* 2010, Jetz *et al.* 2012, Prum *et al.* 2015, Varga *et al.* 2019, Feng *et al.* 2020, Kim *et al.* 2021, Suvorov *et al.* 2022a,b). Despite the newfound abundance of comparative datasets and phylogenies, data that originate from different sources via disparate techniques are not always immediately compatible, and require careful curation prior to subsequent analysis (Marx 2013, Leonelli 2019, Pal *et al.* 2020, Xia *et al.* 2020).

**Figure 1.**
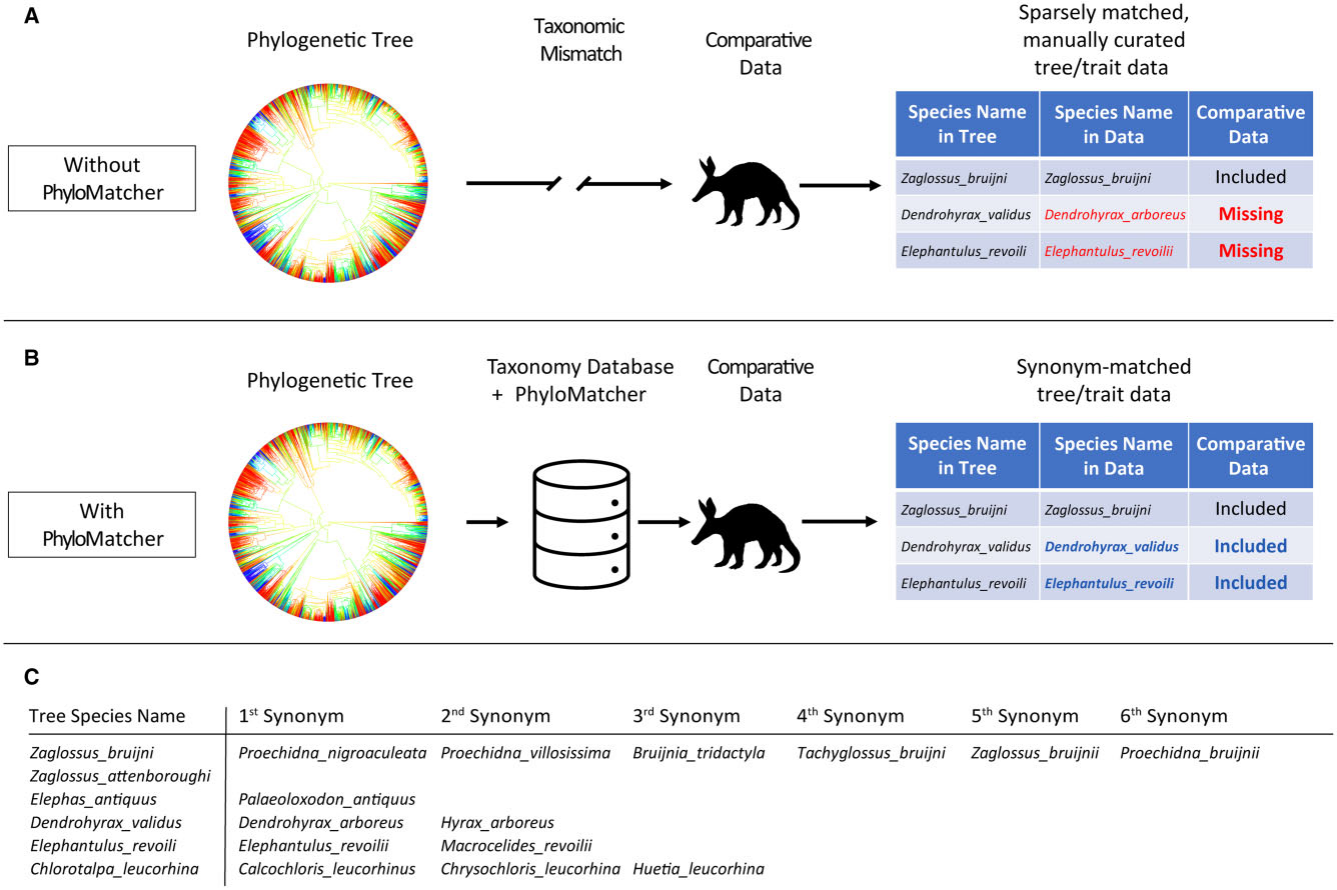
Mismatch of taxonomic nomenclature between species tips in phylogenetic trees and comparative datasets can lead to missing data and taxa being dropped from comparative analysis (A). We present a programmatic tool, PhyloMatcher, that can be used as an intermediary step in comparative workflows (B), to reconcile taxonomic mismatches, increasing the accuracy, completeness, and speed of comparative analyses. PhyloMatcher functions by compiling candidate synonyms for species names from a phylogenetic tree (C), and then reassigning names in the comparative dataset to match names in the tree.

A key necessity for broad studies of macroecology and trait evolution is that comparative data for each taxon must be matched to the appropriate branch tips within phylogenetic trees, which is confounded by mismatches of names between the dataset and the tree (Fig. 1). Taxonomic ambiguity can result from several sources (Boyle *et al.* 2013) including spelling (or orthographic) variants, homotypic synonyms (different names applied to the same type specimen), heterotypic synonyms (different names applied to different type specimens that might represent the same taxon), homonyms (the same name applied to disparate taxa), variation in naming philosophy (e.g. “lumping” versus “splitting” taxa; de Queiroz and Gauthier 1994), and simple misspellings. In addition, taxonomic naming conventions and the accepted names for various taxa have fluctuated through time (e.g. Pauly *et al.* 2009, Eardley and Urban 2006), so many species have accumulated a multitude of synonymous names (Patterson *et al.* 2010, Garnett *et al.* 2020). However, as names have fluctuated, data repositories containing references to those names have remained comparatively stagnant (Schellenberger Costa *et al.* 2023). For example, specimens in natural history research collections have been labeled with these various synonyms through time—an artifact which has been inherited by comparative datasets compiled from the specimens. These collections are vast in both their taxonomic breadth and in the number of samples available for many taxa, facilitating broad comparative studies of a multitude of traits, and because specimens were collected at various times in history, they provide valuable opportunities for time series analyses that would not otherwise be possible (Lister 2011, Holmes *et al.* 2016, Lopez *et al.* 2020, Shultz *et al.* 2021, though this is not without challenges, see Davis *et al.* 2023). However, the use of these collections in a modern phylogenetically informed framework requires that taxonomy of the specimens be reconciled with the taxonomy used when constructing the phylogeny. Similar problems can pervade field-collected datasets. The resulting taxonomic ambiguity poses a serious challenge to phylogenetic comparative analyses. The problem is easily rectified manually with small numbers of taxa, but it becomes less tractable as the number of study taxa increases. In especially taxonomically broad studies, reconciling taxonomic names can impose a serious impediment.

Because of the pervasiveness of mismatched taxonomy throughout biological data, the ability to programmatically reconcile names as an initial step in comparative analyses would be valuable. There are several tools available to fix taxonomic problems in datasets. Taxamatch (Rees 2014) uses fuzzy-matching to reconcile misspellings and typographic errors in taxonomic names, however it is implemented in Oracle PL/SQL, which may limit the breadth of its appeal beyond database administration. Taxize (Chamberlain and Szöcs 2013, Chamberlain *et al.* 2020) is an R package which can query many databases to find correct spellings of a given name, as well as up- and down-stream taxonomic hierarchy. However, it does not have a method for choosing the “best” or canonical name. For instance, searching the misspelled & archaic “*Feliss leo”* is corrected to the archaic name “*Felis leo*”, but not matched to the modern “*Panthera leo*”. Galaxy Tools Taxonmatcher (galaxy-tool-taxonmatcher 2023) is a Python package which can find synonyms between two taxonomy databases but requires manual reconciliation of names in datasets. The Taxonomic Name Resolution Service (TNRS; Boyle *et al.* 2013) matches synonyms in lists of taxonomic names and replaces them with accepted nomenclature via a web interface (https://tnrs.biendata.org/), but it is limited to plant taxonomy. To our knowledge, there are no other freely available utilities or services that match and reconcile synonymous taxonomic names between datasets and phylogenies, and none that operate on taxonomic groups beyond plants. Repositories for naming synonyms already exist: the Global Biodiversity Information Facility (Telenius 2011) and National Center for Biotechnology Information Taxonomy (NCBI; Schoch *et al.* 2020) databases list known current and historical names that have been applied to each taxon. Thus, there are resources available to identify mismatched names, but their use still requires cumbersome manual searching and matching of synonyms to reconcile them with the naming convention used in any given phylogeny.

Here, we present a tool for programmatically matching taxonomic names between datasets: PhyloMatcher, which we have made available as a Python command line tool and a GUI application. We first describe the software’s algorithm (Fig. 1) and then describe how we used PhyloMatcher to reconcile synonymous names for a dataset of mammalian taxonomy with the naming conventions from the phylogenies provided at VertLife.org (Jetz *et al.* 2012, Tonini *et al.* 2016, Jetz and Pyron 2018, Stein *et al.* 2018, Upham *et al.* 2019). We provide a comparison between manual matching of names with the output of PhyloMatcher in both accuracy and speed of matching. We show that our method provides a dramatic reduction in the time needed to link species-level data to phylogenetic trees for comparative analyses, thus overcoming a significant barrier to the analysis of large comparative datasets.

## 2 Methods

The initial implementation of PhyloMatcher draws from two sources (GBIF and NCBI) to populate lists of synonymous taxonomy (Fig. 1). These two databases differ in their structure, so we used different approaches to extract names from each of them. We describe both implementations in the following sections, followed by a brief example of an application of PhyloMatcher.

### 2.1 GBIF implementation

PhyloMatcher uses the species module of the pygbif (Telenius 2011) package as an API client to query GBIF for synonyms. For a given species in the input file, the GBIF backbone database is first queried to retrieve the base-level identity for the target species using the name_backbone function. Upon successful retrieval the current name used by GBIF is stored and the unique species identifier is used to access any known synonyms using the name_usage function. If a “canonical” name is found that is different from the target name, it is returned along with the synonyms and current name. This process is done in parallel using the concurrent.futures package in Python with a user-specified number of threads, four being the default. Once all queries are complete, the results are collated into a CSV file where the first column is the input species name and each successive column is a synonym found in the GBIF database.

### 2.2 NCBI implementation

The NCBI querying module of PhyloMatcher uses the Entrez module (Schuler *et al.* 1996) of the Biopython package (Cock *et al.* 2009) as an API client to query the NCBI Taxonomy database for synonyms. Similar to the GBIF module, the database identifier for a given input species is retrieved using the run_esearch function. Due to the strictness of NCBI’s search, in the case of a failure during this step, a few common letter substitutions are searched (such as swapping “um” for “us” in *Orthriophis taeniurus*, e.g.). Once all possible identifiers have been retrieved, the list is checked for duplicate entries, which are removed. XML entries for all filtered IDs are retrieved and parsed simultaneously using the efetch and read functions. For each entry in the resulting data the “OtherNames” and “Name” fields are exhaustively searched, and all unique entries are returned. Results are collated and output identically to the GBIF module explained above.

### 2.3 Merging the phylogeny with comparative data

Once synonyms have been retrieved a separate module allows for matching comparative data to the phylogenetic tips found in the original data. This module takes in two files: a csv file containing comparative values where the first column is the binomial species name (genus and species, separated by underscores), and the output csv produced by PhyloMatcher’s querying module. The remaining columns in the comparative file can be any data associated with each species such as trait data, occurrence observations, or a multitude of other species-level data to be used in comparative analyses. The synonym lists are stored as a list-of-lists (Fig. 1), and the comparative data are loaded line-by-line. For each line in the comparative file the listed species is checked against the species lists, and if present, is swapped for the name in the phylogeny. A modified version of the comparative data file is written containing a column with the phylogeny-matched species names.

### 2.4 Programmatic versus manual matching

To test the functionality and utility of PhyloMatcher, we matched taxonomic names for a dataset of approximately 24 million occurrence observations of mammals with 15 082 unique species names from the GBIF database with taxonomic names from the mammalian phylogeny from VertLife.org both manually and using PhyloMatcher. The tree contains 5911 of the approximately 6000 extant mammal species (Upham *et al.* 2019), suggesting that there are a large number of taxonomic synonyms present in the occurrence data. We searched the NCBI and GBIF databases for species names in the GBIF mammal occurrence data that do not match tip names in the phylogeny from Vertlife.org (Upham *et al.* 2019). We tracked the time spent manually collecting synonymous names for the mammal dataset, which we compared to the run-time of PhyloMatcher given the same input. We further compared the number of candidate naming synonyms found by each method and the proportion of missing data that was recovered.

## 3 Results

There were 947 out of 5911 (16%) species in the mammalian phylogeny that were not matched by species occurrence observations in the GBIF dataset. Some species were simply not represented in the occurrence data, but others were the product of mismatched taxonomy. Running PhyloMatcher produced 1303 unique candidate naming synonyms, preserving data from 379 species that would have been dropped from subsequent analysis (40% of the missing species). We then replaced synonyms in the occurrence data (∼24 million data points) with names matching the tree, resulting in a reconciled occurrence dataset ready for comparative analysis. The name reconciliation in the occurrence dataset took roughly 600 s of compute time on a single core.

In contrast, our manual effort produced a total of 646 candidate naming synonyms, effectively reconciling 165 species that would have been otherwise dropped from comparative analyses because of mismatched nomenclature (17.4% of the total number of missing data), leading to a loss of 214 species compared to PhyloMatcher. In addition, the manual reconciliation of names took 6.8 h, compared to 107 s of computational time on 8 computing cores for PhyloMatcher for the query step. In summary, PhyloMatcher not only found more candidate synonyms to match taxonomic names in the mammalian occurrence data to the phylogeny than manual matching found and preserved more than double the number of dropped taxa that manual matching was able to, it did so in a small fraction of the time.

## 4 Discussion

Here, we provide a solution to a longstanding challenge to large-scale comparative studies: the problem of mismatched taxonomy (Dayrat 2005, Patterson *et al.* 2010, Garnett *et al.* 2020). Taxonomic conflicts have slowed advances in multiple fields of biology, including phylogeography, evolutionary biology and population genetics, comparative morphology, and biomechanics, and ecology (Dayrat 2005, Bortolus 2008). Efforts to quantify biodiversity (McNeely 2002, Schlick-Steiner *et al.* 2010) and the study of pathogens (especially fungi; Wu *et al.* 2019, Almeida-Silva *et al.* 2021) have been especially impacted by ambiguous taxonomy. We created PhyloMatcher to resolve many of these conflicts. The PhyloMatcher software is available both as a command line program and a GUI application, and provides a programmatic means to reconcile taxonomic names in trait data with the species labels in phylogenetic trees. PhyloMatcher is easy to install and incorporate into existing workflows, and leverages pre-existing and continually maintained databases that include lists of synonymous species names. The input for PhyloMatcher is a list of taxonomic names from a phylogenetic tree and a trait dataset to be matched to it. The output is a new data file with reconciled species names, ready for comparative analyses. In our validation tests, PhyloMatcher roughly doubled the number of candidate matches in our test dataset relative to manual matching, and in a small fraction of the time.

We created PhyloMatcher to assist, narrowly, with the task of matching taxonomic names between comparative data and branch tips of phylogenies. Taxonomy is fraught with complications arising from fluctuating philosophies and practices (Patterson *et al.* 2010, Garnett *et al.* 2020, Schellenberger Costa *et al.* 2023). Indeed, taxonomy differs even among database efforts to catalog and reconcile taxon names (Schellenberger Costa *et al.* 2023). Specimen labels in research collections that have been assembled through time reflect those complexities, hindering analyses that use taxonomic names as the uniting identifiers across datasets. Naming philosophy, which of the synonymous names are generally accepted, and references to particular type specimens (i.e. homotypic versus heterotypic synonyms) are beyond the scope of what PhyloMatcher is designed for. PhyloMatcher takes a pluralistic approach, finding all possible synonyms for each searched name with the expressed goal of utilizing as much of the available data as possible. Of course, this means that some care should be taken in its use. Users are advised to consult recent taxonomic references for their study system to inform decisions regarding appropriate naming standards. Further, careful data hygienics is always a best-practices recommendation. Cross-code homonyms (the same name referring to disparate taxa) may exist among the taxon names in broad comparative datasets, however they should be easily uncovered, appearing as outliers, when looking at almost any trait data.

With the rise of large datasets and taxonomically broad phylogenetic comparative analyses, tools that enhance the integration of multiple data types are increasingly important. Incorporating PhyloMatcher as a simple intermediate step in pre-existing workflows will improve the speed, accuracy, and phylogenetic completeness of comparative biology studies.

## Data Availability

There are no data associated with our manuscript. The software that our manuscript describes is available at the noted github repository, though.
